# Supplementing Tropical Cattle for Improved Nutrient Utilization and Reduced Enteric Methane Emissions

**DOI:** 10.3390/ani9050210

**Published:** 2019-04-30

**Authors:** Asep I. M. Ali, Shimels E. Wassie, Daniel Korir, Lutz Merbold, John P. Goopy, Klaus Butterbach-Bahl, Uta Dickhoefer, Eva Schlecht

**Affiliations:** 1 Animal Husbandry in Tropics and Subtropics, University of Kassel and University of Göttingen, Steinstr. 19, 37213 Witzenhausen, Germany; asepali76@gmail.com; 2 Animal Nutrition and Rangeland Management in the Tropics and Subtropics, Institute of Agricultural Sciences in the Tropics, University of Hohenheim, Fruwirthstr. 31, 70599 Stuttgart, Germany; Shimels_Wassie@uni-hohenheim.de (S.E.W.); uta.dickhoefer@uni-hohenheim.de (U.D.); 3 Mazingira Centre, International Livestock Research Institute (ILRI), 00800 Nairobi, Kenya; D.Korir@cgiar.org (D.K.); L.Merbold@cgiar.org (L.M.); J.Goopy@cgiar.org (J.P.G.); klaus.butterbach-bahl@kit.edu (K.B.-B.); 4 Institute of Meteorology and Climate Research, Atmospheric Environmental Research (IMK-IFU), Karlsruhe Institute of Technology (KIT), 82467 Garmisch-Partenkirchen, Germany

**Keywords:** Boran cattle, greenhouse gas emissions, low-quality roughage, microbial protein synthesis, supplementation, sweet potato vine silage, urea-molasses block

## Abstract

**Simple Summary:**

Quality supplementation of roughage-based cattle diets is recommended to improve the animals’ production in tropical regions. We tested the effects of two widely suggested high-quality low-cost feed supplements on feed intake, nutrient utilization and enteric methane emissions of growing female cattle. While free access to urea-molasses blocks did not effectively improve the key variables, sweet potato vine silage, a by-product of sweet potato cultivation, improved diet digestibility as well as the animals’ retention of nitrogen and lowered their methane emissions per unit of digested feed. Supplementing productive cattle with sweet potato vine silage can thus concomitantly contribute to better animal performance and lower environmental impact.

**Abstract:**

Given their high nitrogen (N) concentration and low costs, sweet potato vine silage (SPVS) and urea-molasses blocks (UMB) are recommended supplements for tropical regions; therefore, they were investigated in this study. Six heifers were allocated to three diets: the roughage diet (R) consisted of wheat straw (0.61) and Rhodes grass hay (0.39; on dry matter (DM) basis); R + SPVS combined R (0.81) and SPVS (0.19); and with R + UMB animals had access to UMB. During two experimental periods, feed intake, feces and urine excretion, digesta passage, and rumen microbial protein synthesis were determined during seven days and methane emissions during three days. There was no treatment effect (*p* > 0.05) on DM and N intake. Apparent DM digestibility of R + SPVS (510 g/kg) was higher (*p* < 0.05) than of R (474 g/kg). Digesta passage and duodenal microbial N flow were similar for all diets (*p* > 0.05), while N retention was highest with R + SPVS (*p* > 0.05). Methane emissions per unit of digested feed (g CH_4_/kg dDM) were lower (*p* < 0.05) for R + SPVS (55.2) than for R (64.7). Hence, SPVS supplementation to poor–quality roughage has the potential to increase diet digestibility and N retention while reducing CH_4_ emissions.

## 1. Introduction

Smallholder mixed crop–livestock systems provide livelihoods for a billion people and produce a third of global beef and milk [1]. These systems are therefore important for local and regional food provision and rural development, especially in tropical countries. However, intensification of cropping activities in mixed systems is often considered more relevant than strengthening of the livestock component [1]. A major concern in this respect is feed supply, because forage production competes with field crops for land, water, labor and other inputs. Consequently, ruminants in mixed systems are often provided with poor-quality forages or crop residues [2], which supply insufficient energy and nutrients [3,4]. This results in low performance [5], live weight (LW) losses [6,7] and a negative nitrogen (N) balance [6,8]. Furthermore, low crude protein (CP) and high fiber concentrations of poor-quality roughages not only entail low feed dry matter (DM) intake and low diet digestibility [9], but also high enteric methane (CH_4_) emissions [10]. In Brahman heifers, for example, the relationship between CH_4_ emissions per unit of digested organic matter (dOM) differed between tropical forages of good and poor quality (64.6 versus 75.4 g CH_4_/kg dOM; [11]). Piñeiro-Vázquez et al. [12] were able to lower CH_4_ emissions when increasing N intake of heifers by supplementing their poor-quality *Pennisetum purpureum* diet with N-rich *Leucaena leucocephala*. Furthermore, improved fiber digestibility and higher N retention were observed as intake of *L. leucocephala* increased [12], most likely due to enhanced microbial N flow from the rumen to the duodenum stimulated by the legume supplementation [13].

Where land and/or water for cultivating leguminous fodder plants is scarce, leafy crop residues, such as sweet potato vines, with high CP and low fiber concentration [14,15,16] may serve as valuable alternative. In many parts of Africa, sweet potato is a regular staple food, even promoted for human diet improvement [17]. Feeding cattle with sweet potato vines is practiced by small-scale farmers in Kenya [3,4] and Nigeria [18]. In dairy cows, this has been shown to improve rumen fermentation, feed digestibility, and milk yield [19], and reduce production costs [18]. Another widely recommended supplement is the urea-molasses block (UMB), due to its easy fabrication and low costs [20]. Increased LW gain with UMB supplementation was reported for grazing dairy heifers in Kenya [21], and improved N retention was determined in Indian cattle consuming wheat straw supplemented with UMB [8,22]. If optimal amounts of readily degradable protein and energy are supplied by the supplement, rumen microbes can more efficiently break down poorly digestible roughages, which reduces enteric CH_4_ production, yields higher microbial biomass, enhances post-ruminal availability of amino acids and increases N retention [22,23,24]. However, most of the cited studies only investigated one or two of the mentioned processes and implications, rather than analyzing the continuum of supplemented feed intake, digesta passage, diet digestibility, rumen microbial protein synthesis, duodenal microbial N flow, N retention and enteric CH_4_ emission. Such a holistic assessment is however needed to identify feeding strategies that serve a sustainable intensification of tropical crop–ruminant systems. We therefore investigated how supplementing a poor-quality roughage diet of dairy heifers with low-cost high-quality supplements, namely sweet potato vine silage (SPVS) and UMB, affects their digestive processes, enteric CH_4_ production and rumen microbial protein synthesis.

## 2. Materials and Methods 

### 2.1. Experimental Design and Animals

An experiment comprising two periods of five weeks each was conducted at the International Livestock Research Institute (ILRI), Nairobi, Kenya, from September to November 2015. The average daily minimum and maximum ambient air temperature during this period was 14.1 °C and 26.0 °C, and relative air humidity ranged from an average minimum of 17.0% to an average maximum of 93.2% (recorded on site with a H08-032-08 HOBO® Temp/RH logger at 0.5 h intervals). Each of the two periods was subdivided into: (i) three weeks of adaptation to the diet; and (ii) two sampling weeks including one week of digestibility measurement where feed intake along with feces and urine excretion were measured and one week of respiration chamber measurements of enteric CH_4_ emissions.

Six Holstein × Boran heifers of 148.0 kg (SD 4.57) initial LW aged 1.3 years were stratified by LW and allocated to three treatments. Crossbreeding local Boran cattle with Holstein Friesian is a common practice in Kenya and neighboring Ethiopia to enhance the milk yield of female individuals. Before starting the experiment, heifers were ear-tagged and subcutaneously vaccinated against foot and mouth disease (inactivated FMD virus strains, 3 mL/animal; Kenya Veterinary Vaccines Production Institute, Nairobi, Kenya) and clostridia (inactivated bacterin-toxoid, 5 mL/animal; Jordan Bio-Industries Center, Amman, Jordan). In addition, they were orally treated against endoparasites (Albendazole 10 g/L; 15 mL/animal orally; NORBROOK Kenya Ltd., Nairobi, Kenya). The heifers were kept in an open barn with individual pens (2.0 m × 3.0 m) during adaptation, in individual stands (1.1 m × 2.2 m) inside a closed barn during digestibility measurements, and in an open circuit chamber (see below) during CH_4_ measurements. Their LW was determined once weekly before morning feeding (Gallagher weigh scale W210; FarmShop, Queensland, Australia; capacity 2000 kg, accuracy 1%). The experiment was approved by the Animal Care and Use Committee of ILRI (No. IACUC-RC2015-07) and the animals were under constant observation of a veterinarian.

### 2.2. Feeding

The experimental diets consisted of roughage (R) only (61.4 g wheat straw and 38.6 g Rhodes grass hay per 100 g diet, on DM basis), roughage and sweet potato vine silage (R + SPVS), and roughage plus urea-molasses block (R + UMB). The composition of the roughage, UMB, and SPVS is shown in Table 1. Straw and hay were chaffed to particles of 5–20 cm length and mixed daily, while the silage was prepared three months before the experiment according to the International Potato Centre’s brochure [25] by mixing 10 kg of molasses, 375 kg of fresh sweet potato vines (leaf and stem), and 175 kg of fresh roots and fermenting the mixture in 1000-liter silo bags. The urea-molasses block contained (g/100 g of fresh matter (FM)): water (5.0), magnesium sulfate (5.0), vegetable oil (1.0), sugarcane molasses (35.0), urea (10.0), sodium chloride (10.0), dicalcium phosphate (18.9), a trace mineral premix (Mn, Zn, Cu, and Se; 0.1), cement (10.0), and cottonseed meal (5.0). 

The amount of roughage offered to each animal corresponded to 2.5 g DM/100 g LW according to the weekly measured LW; it was adjusted to allow for refusals of 5–10 g/100 g (i.e., ad libitum feeding). For diet R + SPVS, roughage contributed 81 g and SPVS 19 g per 100 g (on DM basis) of the offer. During daily feeding, SPVS was offered once in the morning along with a first offer of roughage. To minimize roughage spillage, the daily portion was stored in a large bag and small amounts were offered at a time. When the trough was emptied to two thirds, new roughage was added until the bag was empty. Daily feeding started at 9:30, after refusals from the previous day had been removed from the trough and weighed (Citizen CTG6H scale, Citizen Scales Inc., New York, USA; capacity 6000 g, accuracy 0.1 g). Drinking water and UMB were provided ad libitum and always accessible.

### 2.3. Quantification of Feed Intake, Feces, and Urine Excretion

In each experimental period, approximately 100 g FM of offered roughage was sampled after chaffing and mixing (2 times per week). Likewise, a representative sample of 300 g FM of SPVS was collected from each newly opened silage bag, and UMB (±100 g FM) was sampled at the moment of UMB preparation. Roughage samples were stored in paper bags at room temperature, while samples of SPVS and UMB were stored in zipper bags in a freezer (−20 °C). During the 7-day digestibility measurements, individual refusals of roughage and SPVS were collected and weighed daily. Roughage refusals were stored in a large plastic bag, pooled for the week, and then homogenized and subsampled (±100 g FM) at the end of the week. SPVS refusals were also stored as a pooled sample at −20 °C; they were thawed, homogenized, and subsampled (±100 g DM) at the end of the week. Consumption of UMB was measured by weighing the blocks before each morning feeding, and daily intake was calculated as the weight difference between two subsequent mornings.

Whenever an animal defecated, the total amount of feces was collected from the clean pen floor. Per animal, feces were collected into a 10-liter bucket and weighed (Citizen CTG6H scale, see above) at 8:00 each day throughout the sampling week. After 24 h, feces were thoroughly mixed and a subsample of 300 g FM was dried at 50 °C for 72 h (Genlab SDO/425/DIG oven, Genlab Ltd., Widnes, UK) and reweighed. Another feces subsample of 60 g FM was stored frozen (−20 °C) for N analysis. Dried samples of feed offered, feed refused and of feces were stored in airtight zipper bags at room temperature. At the end of each experimental period, dried samples were ground to pass a 1-mm mesh (IKA® Werke grinder MF 10 basic, Staufen, Germany). Proportional to the daily amount of feces excreted, dried fecal subsamples were pooled per period and homogenized; a final sample of 100 g DM was kept for analysis. Frozen fecal samples were thawed, pooled in proportion to the daily amount of feces excreted, thoroughly mixed and directly analyzed for N (see below).

During five consecutive days of the sampling week, total urine excreted by each heifer was quantitatively collected into a closed 5-L container using a urine harness fitted to the animal’s vulva [26]. Each bucket contained 100 mL of 20% v/v sulfuric acid to acidify the urine (pH < 3) and prevent ammonia losses as well as microbial degradation of purine derivatives. After quantifying the excreted daily volume, urine was homogenized, filtered through two layers of surgical gaze and then sampled, whereby 100 mL were stored at −20 °C for N analysis. For analysis of purine derivatives, 50 mL of acidified urine sample were filtered through a filter paper (DF 400 185, ALBET LabScience, Dassel, Germany). Thereafter, 20 mL of the filtrate were diluted with distilled water at a ratio 1:5 and thoroughly mixed. Three aliquots of 15 mL each were stored at −20 °C for analysis. 

### 2.4. Determination of Digesta Passage

Liquid and solid digesta passage through the gastrointestinal tract were determined using ytterbium (Yb) marked fiber particles (solid digesta marker) and Cobalt-EDTA (Co-EDTA, liquid digesta marker). To prepare marked fiber, wheat straw was hand-cut to >3 cm particle size and then sieved through a 2-cm mesh. Straw remaining on the sieve was boiled for 1 h in neutral detergent solution free of ethylenediaminetetra-acetic acid (EDTA) and then rinsed repeatedly with tap water. Washed fiber particles were dried for 48 h at 70 °C and thereafter soaked for 24 h in a 12.4 mmol/L aqueous solution of ytterbium (III) acetate tetrahydrate. Afterwards, marked fiber was again rinsed with tap water and then soaked for 6 h in an acetic acid solution (100 mmol/L) to discard unabsorbed Yb [27]. After another rinse with tap water, the fiber was dried at 70 °C for 48 h and stored. The final concentration of Yb (8.46 mg/g DM) was determined from a 0.5 g sample of marked fiber (see below).

Co-EDTA marker was prepared according to Uden et al. [28]: 249.08 g cobalt(II) acetate tetrahydrate, 43 g lithium hydroxide, and 292.24 g EDTA were dissolved in a 10-L beaker containing 2 L of Milli-Q distilled water. Hydrogen peroxide (200 mL, 30% v/v) was added to the solution; after overnight equilibration, 3 L of ethanol (95% v/v) were added and the mixture was refrigerated at 4 °C. The resulting precipitate was filtered (Whatman No. 2 filter paper) and washed thoroughly with 80% v/v ethanol. The precipitate was dried overnight at 65 °C and stored in an airtight bag.

Before morning feeding on Day 1 of the sampling week, each heifer was fed a single pulse dose of marked fiber corresponding to 5.6 mg Yb per kg LW [29]. The fiber particles were mixed with 20 g of molasses. As soon as a heifer had completely consumed the marked fiber, it was drenched with Co-EDTA at 23.56 mg/kg LW. The time of marker application (t_0_) was individually recorded as the moment a heifer had been drenched with Co-EDTA. Fecal samples for Yb and Co analysis were collected by gentle anal stimulation and grab-sampling of fresh feces at 0, 4, 6, 8, 10, 12, 14, 16, 20, 24, 28, 32, 36, 40, 46, 52, 58, 64, 70, 76, 82, 88, 96, 104, 112, 120, 128, 136, and 148 h after dosing the markers. Fresh samples were weighed, homogenized, and about 60 g FM were kept for marker determination, while residual material was collected into the 10-liter bucket for daily fecal sampling (see above). Marker-containing samples were dried at 50 °C for 72 h, reweighed, ground to pass a 1-mm mesh (see above), and stored in air-tight zipper bags until analysis. 

### 2.5. Quantification of Enteric Methane Emissions

Methane measurements were carried out in three respiration chambers (3.0 m × 1.5 m, 3 m height; No Pollution Industrial Systems Ltd, Edinburgh, UK) during 22 h on each of 3 days per animal and period, with subsequent measurements separated by one day. At 10:00 on a measurement day, the animal was positioned in the chamber where it was awaited by its feed; drinking water was automatically supplied. At 8:00 on the following day, the animal returned to the open barn and the chamber was cleaned, feed refusals were quantified and feed was placed for the new animal, which again entered at 10:00.

During each 22 h measurement, the concentration of CH_4_ [ppm] in the inlet and outlet air stream was measured every 10 min for 90 s by a Picarro G2508 Cavity Ringdown spectrometer (Piccaro Inc. California, USA). The analyzer was equipped with pumps connected to the incoming and exhaust air streams of the chamber and to two computers that controlled air condition, flow rate, and recorded gas concentrations on a continuous basis. Total CH_4_ emission (g/22 h) was calculated as chamber airflow multiplied by the CH_4_ concentration in the chamber, adjusted for the CH_4_ concentration of the incoming air, temperature, recovery rate, and atmospheric pressure in the chamber, and then converted to daily CH_4_ emission.

### 2.6. Chemical Analysis of Samples

Samples of feed offered and refused and of feces were analyzed for their concentrations of DM (method 967.03; AOAC [30]) and crude ash (method 924.05; AOAC [30]). Concentration of organic matter (OM) was obtained by subtracting the concentration of crude ash (g/100 g DM) from 100. According to VDLUFA [31], neutral detergent fiber (NDF; method 6.5.1, using alpha-amylase) and acid detergent fiber (ADF; method 6.5.2) concentrations—including residual ash—were determined using a FibertecTM FOSS analyser (Foss GmbH; Hamburg, Germany). The N concentration in feedstuffs offered and refused, as well as in thawed feces and urine, was determined by the Kjeldahl procedure (method 988.05; AOAC [30]) using a Tecator 1028 distilling unit (Tecator GmbH; Hagen, Germany); CP concentration was obtained by multiplying N concentration with factor 6.25. All analyses were done in duplicate and repeated if differences between replicates were >5% of the mean.

Marker-containing fecal samples underwent sealed chamber digestion to determine Yb and Co concentrations [32]. Each sample was weighed (0.5 ± 0.01 g DM; Mettler Toledo XP205; Giessen, Germany) and placed in a pre-weighed 100 mL Schott bottle. Two milliliters of a freshly prepared perchloric acid/hydrogen peroxide mixture (7:3 v/v) were placed in the bottle, and contents were allowed to oxidize overnight in a fume cupboard. Then, 1 mL of hydrogen peroxide was added, the bottle was tightly sealed and placed in the oven at 80 °C for 30 minutes. After the bottle had cooled down, another 1 mL of hydrogen peroxide was added, and the tightly sealed bottle was placed in the oven (80 °C) for 60 minutes. Afterwards, the sample was equilibrated to 20 g by addition of distilled water, shaken, filtered to remove silica precipitates (Whatman No. 1 filter paper), and stored at 2 °C until analysis. To check precision of digestion procedure and subsequent spectroscopy, every 10th sample was digested in duplicate. Yb and Co concentrations of the solution (mg/L) were determined by inductively coupled plasma optical emission spectroscopy (ICP-OES 5100 VDV, Agilent Technologies; Santa Clara, USA) after 1:10 dilution with distilled water. Yb and Co concentrations in feces (mg/g DM) were calculated based on dilution, equilibrated weight, and dry sample weight. 

To determine microbial N supply to the host animal, the daily excretion of purine derivatives (PD; allantoin, uric acid, xanthine, and hypoxanthine) was determined by colorimetry [33]. Creatinine was also analyzed colorimetrically using the Jaffe alkaline picrate reaction. The diluted urine samples were thawed, homogenized, and further diluted with distilled water at 1:30 v/v to achieve a total PD concentration in the sample of 10–50 mg/L. Absorbance of allantoin was determined at 522 nm and of uric acid at 293 nm (Shimadzu®UV-150-02 spectrophotometer; Shimadzu Corporation, Kyoto, Japan). Xanthine and hypoxanthine concentrations were derived from uric acid concentration after treating the samples with xanthine oxidases (X1875-5UN; Sigma-Aldrich Chemie GmbH Steinheim, Germany). Allantoin, uric acid, and xanthine plus hypoxanthine excretions (mmol/day) were obtained by multiplying their molar concentrations (mmol/L) with daily urine excretion; adding up the individual values resulted in total PD excretion (mmol/day). 

### 2.7. Data Analysis

Statistical analyses were performed using SAS 9.1 (SAS Institute Inc. Cary, NC, USA); results are presented as arithmetic treatment means and standard error of the mean unless specified otherwise. Feed and nutrient intake was calculated by subtracting an animal’s daily amount of feed refusals (and the nutrients contained therein) from the daily amount of feed (nutrients) offered. The concentration of nutrients in the ingesta, namely the actually consumed feed, was calculated by dividing the amount of a specific nutrient by total feed DM intake. Apparent total tract digestibility (“digestibility”) of feed DM, OM, NDF, and ADF was calculated by subtracting the amount excreted via feces from the respective amount ingested and dividing the difference by the ingested amount.

The cumulative amount of Yb and Co excreted in the sampling week was calculated from the concentration of the respective element in individual fecal samples multiplied by the respective fecal mass at time t_i_ (sampling time). Based on the one-compartment Gamma-2 model of Richter and Schlecht [29], the NLIN procedure (PROC NLIN method = dud) was applied to compute first-time appearance of marker in feces (TT; equivalent to post-ruminal laminar flow), ruminal passage rate (λ), retention time in the mixing compartment (CMRT: 2λ^−1^), and retention time in the total gastrointestinal tract (TMRT: CMRT+TT) for parameters of solid (*s*) and liquid (*l*) digesta passage.

An animal’s nitrogen retention was calculated by subtracting N excretion in feces and urine from N intake. The amount of microbial PD (X) absorbed at the duodenum was calculated from PD excretion (Y) as proposed by Wassie et al. [34]:

Y = 0.85 X + (0.243 LW ^0.75^) (1)

where Y is PD excretion (mmol/day), X is the amount of microbial PD absorbed at the duodenum (mmol/day), and LW (kg) is the animal’s live weight. Factor 0.243 represents the average daily excretion of endogenous PD in zebu cattle [34].

Duodenal microbial N flow (g/d; also referred to as “microbial N”) was estimated from absorbed PD (X in Equation (1)) as follows:

Microbial N = X * 70 / (0.116 * 0.83 * 1000)(2)

where X is the duodenal absorption of microbial PD (mmol/day), 70 is the N concentration of purines (mg N/mmol), 0.83 is the intestinal digestibility of microbial purines, and 0.116 is the ratio of purine N to total N in mixed rumen microbes. The efficiency of microbial protein synthesis (EMPS) was expressed in three different way, namely as g microbial N (for calculation see Equation (2)) per unit of ingested OM (g N/kg OM), ingested N (g N/g N), and digested OM (g N/kg dOM).

Data from 2 periods × 3 treatments × 2 animals were obtained for feed intake, digestibility, digesta passage parameters, N retention, microbial N flow, and CH_4_ emissions. Analysis of variance (PROC MIXED) was performed with diet and period as fixed effects and animal as random factor:

y_ijk_ = *µ* + *d*_i_ + *p*_j_ + *dp*_ij_ + *a*_k_ + *e*_ijkl_ (3)

where y_ijk_ is the dependent variable for a particular ijk case, *µ* is the overall mean, *d*_i_ is the effect of diet i, *p*_j_ is the effect of period j, *a*_k_ is the random effect of animal k, and *e*_ijkl_ is the residual error.

The Tukey post-hoc test was applied to detect significant differences between diet and period means, respectively. Significance was declared at *p* ≤ 0.05, and a trend was considered to exist if 0.05 < *p* ≤ 0.10. The relationship between feed intake, ingesta quality and rate of passage parameters was tested by Spearman rank correlation (PROC CORR). Linear regression (PROC REG) was used to test the relationship between DM intake (DMI) and daily CH_4_ emission. 

## 3. Results

### 3.1. Feed Intake, Ingesta Composition, Digestibility, and Digesta Passage

The offered roughage was characterized by high NDF and ADF concentrations and a low CP content, whereas SPVS and UMB were low in NDF and ADF and high in CP (Table 1). Even though for all nutrients highest intake values (g/kg^0.75^ LW) were determined for R + SPVS, there was no effect of diet on the quantitative intake of DM, OM, NDF, ADF, and CP (Table 2). Ingesta CP concentration was higher for R + SPVS than for R + UMB and R. Dry matter digestibility was higher for R + SPVS than for R but not different from R + UMB, while OM digestibility of R + SPVS tended to be higher than of R and R + UMB. No difference between diets existed for NDF and ADF digestibility (Table 2). Among all observed variables, only NDF intake was influenced by experimental period (*p* = 0.04) with the value in Period 2 being higher than in Period 1. A significant diet × period interaction was also obtained for NDF digestibility (*p* =0.049).

There were no differences in liquid and solid digesta passage parameters between diets or periods, respectively (Table 3). Spearman correlation analysis indicated that ingesta ADF concentration was negatively correlated (Table 4) with ruminal outflow rate λ and positively correlated with CMRT of solid but not of liquid digesta. Quantitative intake (g/kg^0.75^ LW) of DM, OM, CP, NDF, and ADF was positively related to λ*s* and negatively to CMRT*s* and TMRT*s*. Furthermore, NDF and ADF digestibility showed a positive correlation with λ*s* and a negative correlation with CMRT*s*, and TMRT*s*. Among the liquid digesta passage parameters, only TT*l* showed a negative correlation with OM as well as ADF digestibility (Table 4).

### 3.2. Nitrogen Balance and Efficiency of Microbial Protein Synthesis

Nitrogen intake with R + SPVS was 20% higher than with R and R + UMB, due to higher ingesta CP (and hence N) concentration (*p* = 0.04). However, diet had no effect on microbial N flow, the efficiency of its synthesis (EMPS) and N balance (Table 5). Nitrogen retention was negative for all treatments but tended to be highest with R + SPVS (*p* = 0.051). This was in line with daily LW losses of −0.50, −0.25, and −0.30 kg/d for R, R + SPVS, and R + UMB across the two periods (data not shown). Concentration of total PD and creatinine in urine, total PD excretion, and duodenal microbial N flow (Table 5) were highest in R + SPVS (*p* > 0.10). Microbial N flow correlated positively with λ*l* and negatively with CMRT*l* and TMRT*l* (Table 4). There was no effect of experimental period on N balance and microbial protein synthesis. 

### 3.3. Methane Emissions

There was no difference between diets when relating CH_4_ emission to feed intake (Table 6), irrespective of the constituent (i.e., DM, OM, NDF, and ADF). Methane emission per unit of digested (d) DM (dDM) was lower in R + SPVS than in R (*p* = 0.041) but similar to R + UMB. In addition, there was a tendency (*p* = 0.09) towards lower CH_4_ emission per unit of dOM in R + SPVS (Table 6), whereas CH_4_ emitted per unit of dNDF and dADF was only numerically lower for R + SPVS than for the other diets (*p* > 0.10). Daily emission (g CH_4_/animal·day) correlated significantly with liquid digesta passage parameters λ*l,* CMRT*l* and TMRT*l* (Table 7), as well as with TMRT*s*, whereas for λ*s* and CMRT*s* there was only a weak correlation (r_s_ < 0.56). In contrast, CH_4_ yield, that is g CH_4_ per kg DMI, strongly correlated with solid digesta passage parameters (λ*s,* CMRT*s*, TMRT*s*). A significant correlation with solid digesta passage parameters also existed for CH_4_ emitted per unit of ingested OM and NDF, and for CH_4_ emitted per unit of dDM, dOM, and dNDF (Table 7). Increasing DMI increased daily CH_4_ emission (R^2^ = 0.62, *p* = 0.003) but decreased CH_4_ yield (R^2^ = −0.68, *p* = 0.008).

## 4. Discussion

### 4.1. Supplementation Effects on Intake, Digestibility, and Digesta Passage

Supplementation of SPVS and UMB to the roughage had no effect on feed intake, despite lower NDF and ADF and higher CP concentrations in both supplements, and only SPVS supplementation increased ingesta CP concentration. The lack of intake improvement through SPVS and UMB supplementation might be explained by a limited improvement of feed degradation in the rumen as the basal diet (R) already contained 79 g CP/kg DM. Similar DM and OM intakes of basal and supplemented diet were also reported by Piñeiro-Vázquez et al. [12] for heifers on *P. purpureum* (71 g CP, 659 g NDF per kg DM) supplemented with *Leucaena leucocephala*. However, at lower CP concentrations of the basal diet, an increased intake and digestibility (of DM, OM, CP, NDF, and ADF) was observed in growing goats feeding on *Ischaemum aristatum* (68 g CP, 396 g NDF per kg DM) and supplemented with fresh sweet potato vine [35], and in Boran steers fed wheat straw (20 g CP, 807 g NDF per kg DM) supplemented with *Calliandra calothyrsus* leaves [36].

The CP concentration in the present SPVS was within the range of CP concentrations in leaves and stems of 15 sweet potato varieties from Vietnam [14], but higher than reported from Nigeria [18], and lower than reported from Ethiopia [16] and Kenya [15]. Present NDF and ADF concentrations of SPVS were higher than reported by these four studies. Such differences in SPVS composition might be due to differences in variety, stage of growth, harvest season, and way of silage preparation.

Higher CP and lower NDF and ADF concentrations in ingesta of R + SPVS as compared to R and R + UMB improved dDM and by trend also dOM and dNDF, but had no effect on dADF. The lack of effects of UMB supplementation on digestibility of diet constituents might be partly explained by its low daily intake that averaged 51 g DM (± 11.0), equivalent to 1.8 g DM per 100 g DMI and 8.3 g N per 100 g N intake. Difficulties in the exact determination of daily UMB intake were encountered, even though an unused lick block served as reference to account for weight differences in the hygroscopic material due to changing air humidity.

No significant correlation was found between liquid digesta passage parameters and intake. However, increasing intake increased the hourly outflow of particles from the rumen (λ*s*) and reduced rumen and total tract retention time of solid digesta (CMRT*s*, TMRT*s*). In contrast to this, Mlay et al. [37] reported that passage rate of particles was not modified by increasing intake of heifers fed hay and supplemented with either urea or soybean cake. The fact that λ*l* was not related to feed intake in the present experiment may be due to the high NDF concentration of ingesta: for cattle on fiber-rich diets, Das and Singh [38] observed higher rumen solid and liquid contents and a higher DM pool size with increasing DMI due to supplementation. Moreover, dietary fiber content positively correlates with its water-holding capacity and increases liquid volume in the rumen [39]. 

### 4.2. Supplementation Effects on Nitrogen Balance and Microbial Protein Synthesis

There was a tendency towards higher N retention in heifers consuming R + SPVS, due to higher ingesta N concentration and greater DM digestibility. Several studies reported high protein quality of sweet potato leaves, with a good N solubility and with essential amino acids accounting for 40.7% of total CP [40,41]. Even though N balance was negative in the present study, its improvement through SPVS supplementation agrees with reports on higher N intake and retention in Boran steers fed wheat straw supplemented with *C. calothyrsus* as compared to a pure wheat straw diet [36]. Improved N balance and higher LW gain was also obtained for cattle on wheat straw supplemented with UMB [8]. Piñeiro-Vázquez et al. [12] reported that increasing legume supplementation increased N intake and N retention of heifers despite higher urine N excretion and similar fecal N excretion. An improved N balance along with higher rumen ammonia and total VFA concentrations and a higher yield of bacteria per mol ATP was reported for cattle on wheat straw supplemented with three different UMB formulas [22]. Abdulrazak et al. [42] found that urine PD excretion ranged from 68.6 to 81.9 mmol/day in grass-fed steers and increased with legume supplementation, but microbial N flow to the duodenum and EMPS remained unaffected. In contrast, urine PD excretion of Boran steers on wheat straw supplemented with *C. calothyrsus* was only 7.9–13.6 mmol per 100 kg LW [36] and thus lower than the present average values (28, 32, and 25 mmol per 100 kg LW for R, R + SPVS, and R + UMB). Furthermore, for all diets tested in the current study, EMPS per unit of dOM (16.98–21.61 g N/kg dOM) was higher than the values (−3.00–1.75 g N/kg dOM) reported for the Boran steers [36]. The lack of differences in PD excretion and thus microbial N flow between the tested diets agrees with findings of Phesatcha and Wanapat [19] for a diet of urea-treated rice straw with or without concentrate supplementation, even though their absolute values of PD excretion (165–179 mmol/day) were higher than the present ones (36–50 mmol/day).

The 7% higher outflow rate of solids (λ*s*) with R + SPVS as compared to R likely reduced the proportion of feed nutrients fermented in the rumen and thus counteracted the increased substrate supply to rumen microbes due to higher feed intake and digestibility. In this line, faster rumen passage of solid digesta might have increased the availability of undegraded feed CP for post-ruminal digestion and absorption by the host animal and thus N retention. An increase of duodenal microbial N flow with greater feed intake is reflected by the correlation between microbial N flow and λ*l* as well as CMRT*l*, while the absence of differences in microbial N flow and EMPS between the three diets indicates that SPVS and UMB supplementation did not enhance microbial growth in the rumen. This might in part be explained by a sufficiency of rumen degradable protein at a DM concentration of 7.9% CP in the roughage [24,43]. 

### 4.3. Supplementation Effects on Enteric Methane Emissions

**Figure 1:**
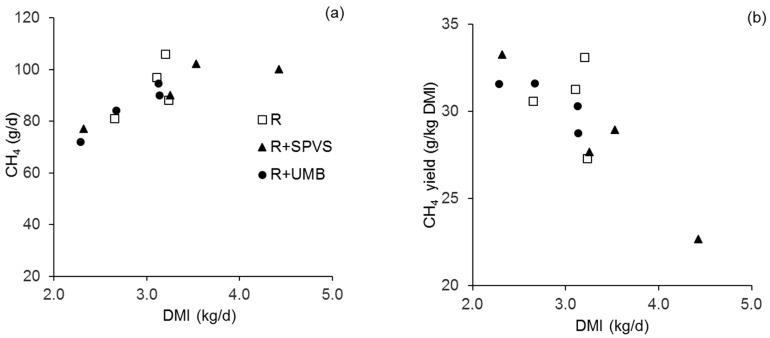
Relationship of the animals’ daily dry matter intake (DMI) to: (**a**) methane (CH_4_) emissions (R^2^ = 0.62); and (**b**) CH_4_ yield (R^2^ = 0.68). The legend in (**a**) also applies to (**b**). R, roughage (0.61 wheat straw + 0.39 Rhodes grass hay); SPVS, sweet potato vine silage: UMB, urea-molasses block; DMI, dry matter intake.

Across diets, CH_4_ yield was within the range of 25.7–31.9 g CH_4_/kg DMI determined in Holstein cows fed a mixed alfalfa/corn silage [44]. Present CH_4_ yields were however greater than the 17.3–22.4 g CH_4_/kg DMI determined for steers fed tropical grasses ad libitum [45], and the 22.6 g CH_4_/kg DMI reported for Holstein steers on barley silage and steam-rolled barley [46]. Increasing DMI (kg/day) increased CH_4_ emission (g CH_4_/animal·day) and decreased CH_4_ yield (Figure 1), illustrating the influence of feed intake on energy loss via CH_4_. Reduced CH_4_ yields were also reported for tropical [47] and temperate [48] forages and cattle breeds when intake increased.

Generally, CH_4_ yield positively correlates with dietary fiber concentration due to greater hydrogen yields when higher proportions of acetate are formed in the rumen [10,49]. The high NDF and ADF concentrations in diets R and R + UMB increased rumen retention time of particles [50,51], thereby prolonging time available for CH_4_ formation [49]. Present CH_4_ emission only correlated with λ*l* but not with any other digesta passage parameter. Goopy et al. [52] also reported that CH_4_ yield was more strongly associated with CMRT*l* (R² = 0.69) than with CMRT*s* (R² = 0.56). In sheep fed several levels of ryegrass, differences in CH_4_ yield were better explained by λ*l* than by λ*s*, because quantitative intake had a stronger effect on rumen passage of liquid than of solid digesta, with increasing intake decreasing CMRT*l*, CMRT*s*, TMRT*l*, and TMRT*s* as well as CH_4_ yield [48]. Likewise, in the present study, increasing intake decreased CMRT*s,* TMRT*s* and CH_4_ yield. The significant relationship between λ*s* and CMRT*s* with CH_4_ per unit of DMI and dDM underlines the strong dependency of enteric CH_4_ formation on rumen digesta kinetics.

A certain potential for CH_4_ mitigation by upgrading a poor-quality roughage diet was demonstrated for SPVS supplementation, which reduced CH_4_ emission per unit dDM. Although SPVS supplementation only slightly improved dDM and had a significant effect on neither rumen microbial protein synthesis (indicator for the activity of rumen bacteria [33]) nor on the ruminal passage rate of solid digesta (λ*s*), the respective values were numerically higher for R + SPVS than for R and R + UMB. The absence of effects of UMB supplementation might be due to the low UMB intake as discussed above. The slight increase in N intake with R + SPVS improved the animals’ N retention with no decrease of N excretion via feces and urine. The latter points to an excess of rumen ammonia in the SPVS diet which should be balanced by supplying additional energy [23,24]. The unchanged nitrogen excretion in the present study also indicates that an evaluation of feeding strategies that mitigate greenhouse gas emissions from ruminants should also consider if and how a reduction of enteric CH_4_ formation affects emissions from excreta, such as nitrous oxide and ammonia [53,54].

## 5. Conclusions

SPVS and UMB supplementation of poor quality roughage did not improve feed intake of Holstein × Boran heifers. However, enhanced ingesta CP content with SPVS supplementation improved feed digestibility and reduced formation of enteric CH_4_ per unit of dDM. Neither supplement enhanced the efficiency of microbial protein synthesis, but SPVS decreased rumen retention time of solid digesta and positively affected N balance. Therefore, supplementing cattle on poor quality roughage with SPVS has the potential to improve diet digestibility, N retention, and mitigate greenhouse gas emissions in tropical smallholder systems, in particular in the end of the dry season when forages are scarce and fibrous.

## Figures and Tables

**Table 1:** Proximate composition of roughage ^1^, sweet potato vine silage (SPVS), and urea-molasses blocks (UMB) offered during the experiment.

Feed	Period ^2^	DM	OM	CP	NDF	ADF
		(g/kg FM)	(g/kg DM)			
Roughage ^1^	1	813	889	70.3	731	479
	2	742	895	72.2	738	460
SPVS	1	193	875	136.1	537	393
	2	199	877	143.7	537	393
UMB	1, 2	899	502	373.6	27	17

DM, dry matter; OM, organic matter; CP, crude protein; NDF, neutral detergent fiber; ADF, acid detergent fiber; FM, fresh matter ^1^ Roughage: 0.61 wheat straw + 0.39 Rhodes grass hay. ^2^ Period 1: 7 September–11 October 2015; Period 2: 12 October–15 November 2015.

**Table 2:** Quantitative intake, ingesta composition, and digestibility of proximate nutrient and fiber fractions in heifers fed roughage (R) ^1^ alone or supplemented with sweet potato vine silage (R + SPVS) or urea-molasses blocks (R + UMB).

Variable	R	R + SPVS	R + UMB	SEM	*p*-Value
Daily intake (g/kg^0.75^ LW) and (share of supplement, g/100 g)					
DM	70.9	76.0 (19.3)	66.8 (1.7)	3.08	0.170
OM	63.1	67.5 (19.0)	59.0 (1.0)	2.79	0.158
NDF	50.4	52.6 (15.0)	47.7 (0.1)	2.22	0.230
ADF	32.3	33.9 (17.0)	30.2 (0.1)	1.30	0.311
CP	5.6	6.7 (30.5)	5.5 (7.7)	0.31	0.113
Ingesta composition (g/kg DM)					
OM	890	887	884	2.21	0.129
NDF	709	691	715	9.31	0.122
ADF	456	447	453	3.62	0.163
CP	79 ^a^	88 ^b^	82 ^a^	0.21	0.040
Digestibility (g/kg)					
DM	474 ^a^	510 ^b^	480 ^ab^	10.07	0.041
OM	509 ^A^	539 ^B^	512 ^AB^	7.78	0.071
NDF	496 ^A^	530 ^B^	506 ^AB^	8.28	0.058
ADF	428	458	429	6.79	0.134

^1^ R, roughage (0.61 wheat straw + 0.39 Rhodes grass hay); DM, dry matter; OM, organic matter; CP, crude protein; NDF, neutral detergent fiber; ADF, acid detergent fiber; SEM, standard error of the mean; LW, live weight. *p*-values for independent variable diet obtained with PROC MIXED. Within rows, means with different lowercase superscripts differ at *p* ≤ 0.05, means with different uppercase superscripts differ at 0.05 < *p* ≤ 0.10 (Tukey post-hoc test).

**Table 3:** Parameters of liquid (*l*) and solid (*s*) digesta passage in heifers fed roughage (R) ^1^ alone or supplemented with sweet potato vine silage (R + SPVS) or urea-molasses blocks (R + UMB).

Variable	R	R + SPVS	R + UMB	SEM	*p*-Value
Liquid digesta passage					
λ*l* (%/h)	9.2	9.2	9.0	0.43	0.972
TT*l* (h)	4.3	3.1	4.4	0.39	0.230
CMRT*l* (h)	22.0	22.0	23.2	1.08	0.854
TMRT*l* (h)	26.3	25.0	27.6	0.97	0.567
Solid digesta passage					
λ*s* (%/h)	3.5	3.8	3.4	0.14	0.262
TT*s* (h)	18.9	16.8	16.2	0.77	0.368
CMRT*s* (h)	56.8	54.4	60.2	2.08	0.298
TMRT*s* (h)	75.8	71.2	76.4	2.08	0.460

^1^ R, roughage (0.61 wheat straw + 0.39 Rhodes grass hay); λ, ruminal passage rate; TT, post-ruminal transit time; CMRT, retention time in the rumen; TMRT, retention time in total gastrointestinal tract; SEM, standard error of the mean. *p*-values for independent variable diet obtained with PROC MIXED.

**Table 4:** Spearman correlation coefficients (r_s_) and significance levels ^1^ of the individual relationships between quantitative intake, ingesta concentration and digestibility of organic matter (OM), crude protein (CP), neutral detergent fiber (NDF), acid detergent fiber (ADF), duodenal microbial nitrogen (N) flow, and efficiency of rumen microbial protein synthesis (EMPS) with liquid (*l*) and solid (*s*) ruminal passage rate (λ), post ruminal transit time (TT), rumen retention time (CMRT) and total tract retention time (TMRT) in heifers fed with roughage alone or supplemented with sweet potato vine silage and urea-molasses blocks.

Variable	Liquid Digesta Passage								Solid Digesta Passage							
	λ*l* (%/h)		TT*l* (h)		CMRT*l* (h)		TMRT*l* (h)		λ*s* (%/h)		TT*s* (h)		CMRT*s* (h)		TMRT*s* (h)	
Feed intake (g/kg^0.75^ LW)																
OM	0.29	^n.s.^	−0.36	^n.s.^	−0.29	^n.s.^	−0.57	^(*)^	0.92	^***^	0.45	^n.s.^	−0.92	^***^	−0.75	^**^
CP	0.42	^n.s.^	−0.26	^n.s.^	−0.42	^n.s.^	−0.69	^*^	0.82	^**^	0.31	^n.s.^	−0.82	^**^	−0.66	^*^
NDF	0.29	^n.s.^	−0.29	^n.s.^	−0.29	^n.s.^	−0.52	^(*)^	0.95	^***^	0.48	^n.s.^	−0.95	^***^	−0.78	^**^
ADF	0.31	^n.s.^	−0.36	^n.s.^	−0.31	^n.s.^	−0.60	^*^	0.91	^***^	0.45	^n.s.^	−0.91	^***^	−0.74	^**^
Ingesta composition (g/kg DM)																
OM	−0.26	^n.s.^	−0.31	^n.s.^	0.26	^n.s.^	0.05	^n.s.^	0.45	^n.s.^	0.57	^(*)^	−0.45	^n.s.^	−0.33	^n.s.^
CP	0.25	^n.s.^	−0.20	^n.s.^	−0.25	^n.s.^	−0.48	^n.s.^	0.31	^n.s.^	−0.15	^n.s.^	−0.31	^n.s.^	−0.30	^n.s.^
NDF	−0.14	^n.s.^	−0.06	^n.s.^	0.14	^n.s.^	0.21	^n.s.^	0.21	^n.s.^	0.08	^n.s.^	−0.21	^n.s.^	−0.21	^n.s.^
ADF	−0.55	^(*)^	0.07	^n.s.^	0.55	^(*).^	0.65	^*^	−0.70	^*^	−0.19	^n.s.^	0.70	^*^	0.57	^(*).^
Digestibility (g/kg)																
DM	0.13	^n.s.^	−0.62	^*^	−0.13	^n.s.^	−0.41	^n.s.^	0.65	^*^	−0.10	^n.s.^	−0.65	^*^	−0.73	^**^
OM	0.00	^n.s.^	−0.70	^*^	0.00	^n.s.^	−0.31	^n.s.^	0.45	^n.s.^	−0.17	^n.s.^	−0.45	^n.s.^	−0.55	^(*)^
NDF	0.20	^n.s.^	−0.55	^(*)^	−0.20	^n.s.^	−0.43	^n.s.^	0.64	^*^	−0.11	^n.s.^	−0.64	^*^	−0.76	^**^
ADF	0.10	^n.s.^	−0.69	^*^	−0.10	^n.s.^	−0.37	^n.s.^	0.68	^*^	−0.02	^n.s.^	−0.68	^*^	−0.76	^**^
Duodenal microbial N flow (g N/day)	0.66	^*^	−0.04	^n.s.^	−0.66	^*^	−0.76	^**^	0.48	^n.s.^	0.00	^n.s.^	−0.48	^n.s.^	−0.53	^(*)^
EMPS																
(g N/kg OM intake)	0.42	^n.s.^	0.09	^n.s.^	−0.42	^n.s.^	−0.39	^n.s.^	0.01	^n.s.^	−0.02	^n.s.^	−0.01	^n.s.^	−0.07	^n.s.^
(g N/kg dOM)	0.42	^n.s.^	0.09	^n.s.^	−0.42	^n.s.^	−0.39	^n.s.^	0.01	^n.s.^	−0.02	^n.s.^	−0.01	^n.s.^	−0.07	^n.s.^
(g N/g N intake)	0.28	^n.s.^	−0.01	^n.s.^	−0.28	^n.s.^	−0.21	^n.s.^	−0.03	^n.s.^	0.06	^n.s.^	0.03	^n.s.^	−0.06	^n.s.^

DM, dry matter; OM, organic matter; dOM, digested OM; CP, crude protein; N, nitrogen; NDF, neutral detergent fiber; ADF, acid detergent fiber; LW, live weight; EMPS, efficiency of microbial protein synthesis, Significance levels, n.s. non-significant (*) *p* ≤ 0.10, * *p* ≤ 0.05, ** *p* ≤ 0.01, *** *p* ≤ 0.001.

**Table 5:** Nitrogen (N) balance, excretion of purine derivatives (PD), duodenal microbial N flow, and efficiency of rumen microbial protein synthesis (EMPS) in heifers fed with roughage (R) ^1^ alone or supplemented with sweet potato vine silage (R + SPVS) and urea-molasses blocks (R + UMB).

Variable	R	R + SPVS	R + UMB	SEM	*p* Value
Nitrogen balance (g/kg^0.75^ LW)					
N intake	0.90	1.08	0.88	0.050	0.113
Fecal N excretion	0.60	0.65	0.60	0.028	0.367
Urine N excretion	0.45	0.48	0.45	0.026	0.655
N retention	−0.15 ^A^	−0.05 ^B^	−0.17 ^A^	0.021	0.051
Fecal-to-urine N ratio	1.33	1.44	1.32	0.066	0.576
Urine PD excretion (mmol/day)					
Allantoin	35.62	40.55	29.62	2.524	0.281
Uric acid	7.21	9.87	6.07	0.849	0.324
Xanthine + hypoxanthine	0.03	0.06	0.02	0.006	0.150
Total PD excretion	42.86	50.48	35.72	3.274	0.283
Creatinine (mmol/day)	29.66	33.63	25.70	1.357	0.452
PD-to-creatinine ratio	1.45	1.50	1.38	0.078	0.572
Duodenal microbial N flow (g/day)	27.73	33.95	21.85	2.696	0.512
EMPS					
(g N/kg OM intake)	10.23	11.63	8.76	0.849	0.139
(g N/kg dOM)	20.20	21.61	16.98	1.601	0.617
(g N/g N intake)	0.71	0.73	0.59	0.052	0.126

^1^ R, roughage (wheat straw + Rhodes grass hay); LW, live weight; OM, Organic matter; dOM, digested OM; SEM, Standard error of the mean. *p*-values for independent variable diet obtained with PROC MIXED. Within rows, means with different uppercase superscript differ at 0.05 < *p* ≤ 0.01 (Tukey post-hoc test).

**Table 6:** Methane (CH_4_) emissions by heifers fed with roughage (R) ^1^ alone or supplemented with sweet potato vine silage (R + SPVS) or urea-molasses blocks (R + UMB).

Methane (g) Emitted	R	R + SPVS	R + UMB	SEM	*p*-value
/animal·day	93.1	92.4	85.3	3.00	0.611
/kg DMI	30.6	28.1	30.6	0.85	0.240
/kg OMI	34.4	31.7	34.6	0.99	0.238
/kg NDFI	43.2	40.8	42.7	1.33	0.538
/kg ADFI	67.0	62.9	67.4	1.76	0.395
/kg dDM	64.7 ^b^	55.2 ^a^	63.8 ^ab^	2.33	0.041
/kg dOM	67.8 ^B^	58.9 ^A^	67.5 ^B^	2.28	0.094
/kg dNDF	88.1	77.0	84.6	3.52	0.167
/kg dADF	157.5	137.7	157.1	5.40	0.133

^1^ R, roughage (0.61 wheat straw + 0.39 Rhodes grass hay); DM, dry matter; OM, organic matter; NDF, neutral detergent fiber; ADF, acid detergent fiber; DMI, DM intake; OMI, OM intake; NDFI, NDF intake; ADFI, ADF intake; dDM, digested DM; dOM, digested OM; dNDF, digested NDF; dADF, digested ADF; SEM, standard error of the mean. *p*-values for independent variable diet obtained with PROC MIXED. Within rows, means with different lowercase superscripts differ at *p* ≤ 0.05; means with different uppercase superscripts differ at 0.05 < *p* ≤ 0.01 (Tukey post-hoc test)

**Table 7:** Spearman correlation coefficients (r_s_) and significance levels ^1^ of the relationship between liquid (*l*) and solid (*s*) digesta passage parameters and the emission of methane in heifers fed with roughage alone or supplemented with sweet potato vine silage or urea-molasses blocks.

Parameter	Methane (g) emitted													
	/Animal·Day		/kg DMI		/kg OMI		/kg NDFI		/kg dDM		/kg dOM		/kg dNDF	
Liquid digesta passage														
λ*l* (%/h)	0.78	^**^	−0.13	^n.s.^	−0.15	^n.s.^	−0.15	^n.s.^	−0.14	^n.s.^	−0.06	^n.s.^	−0.16	^n.s.^
TT*l* (h)	0.20	^n.s.^	0.39	^n.s.^	0.34	^n.s.^	0.39	^n.s.^	0.54	^(*)^	0.57	^(*)^	0.52	^(*)^
TMRT*l* (h)	−0.84	^***^	0.40	^n.s.^	0.41	^n.s.^	0.41	^n.s.^	0.43	^n.s.^	0.36	^n.s.^	0.43	^n.s.^
Solid digesta passage														
λ*s* (%/h)	0.55	^(*)^	−0.90	^***^	−0.91	^***^	−0.90	^***^	−0.84	^***^	−0.81	^**^	−0.83	^***^
TT*s* (h)	0.03	^n.s.^	−0.41	^n.s.^	−0.40	^n.s.^	−0.28	^n.s.^	−0.16	^n.s.^	−0.22	^n.s.^	−0.04	^n.s.^
TMRT*s* (h)	−0.62	^*^	0.68	^*^	0.70	^*^	0.75	^**^	0.76	^**^	0.67	^*^	0.84	^***^

^1^ Significance levels: n.s., not significant, (*) *p* ≤ 0.10, *p ≤ 0.05, **p ≤ 0.01, ***p ≤ 0.001; DMI, dry matter intake; OMI, organic matter intake; NDFI, neutral detergent fiber intake; dDM, digested dry matter; dOM, digested organic matter; dNDF, digested NDF; λ, ruminal passage rate, TT, post ruminal transit time; TMRT, retention time in total gastrointestinal tract.
